# Educational attainment and employment status in lung cancer patients: a nationwide study

**DOI:** 10.1007/s12094-025-03994-y

**Published:** 2025-07-09

**Authors:** Morten Borg, Anders Løkke, Margrethe Henriksen, Sara Witting Christensen Wen, Rikke Ibsen, Ole Hilberg

**Affiliations:** 1 Department of Medicine, Lillebaelt Hospital Vejle, University Hospital of Southern Denmark, Beriderbakken 4, 7100 Vejle, Denmark; 2 Department of Regional Health Research, University of Southern Denmark, Campusvej 55, 5230 Odense M, Denmark; 3 Department of Oncology, Lillebaelt Hospital Vejle, University Hospital of Southern Denmark, Beriderbakken 4, 7100 Vejle, Denmark; 4 i2 Minds, Nørrebrogade 18B, 8000 Aarhus C, Denmark

**Keywords:** Lung cancer, Socioeconomic disparities, Educational attainment, Employment status, Screening eligibility

## Abstract

**Purpose:**

Lung cancer remains the leading cause of cancer-related deaths globally, with low-dose computed tomography (LDCT) screening widely implemented in several countries. However, current screening eligibility, based largely on the National Lung Cancer Screening Trial and NELSON trial outcomes, does not account for socioeconomic risk factors such as education and employment status, which may impact lung cancer incidence and outcomes.

**Methods and patients:**

This study used data from the Danish National Patient Register to examine educational attainment and employment status among 109,940 lung cancer patients diagnosed between 1994 and 2018, compared to a randomly selected matched general population cohort.

**Results and conclusions:**

Patients were less likely to hold higher education degrees and more likely to be on disability pension compared to controls, underscoring a significant socioeconomic disparity. Lung cancer patients frequently had only primary school education, with this educational gap widening over time. Employment disparities were also noted, with lung cancer patients twice as likely to be on disability benefits. These findings suggest that socioeconomic vulnerabilities, including educational level and employment, may exacerbate lung cancer risk, possibly linked to higher smoking prevalence and occupational carcinogen exposure in lower socioeconomic groups. Targeted public health initiatives focusing on smoking cessation and LDCT access for socioeconomically disadvantaged individuals are crucial for addressing these disparities and improving lung cancer outcomes. National wealth alone appears insufficient in bridging these socioeconomic gaps, emphasizing the need for strategic, targeted interventions in lung cancer prevention and early detection.

## Introduction

The social determinants of health, such as education, income, and employment, are increasingly recognized as important factors in lung cancer risk and outcomes. Lower socioeconomic status has been associated with increased lung cancer incidence and worse prognosis, even after controlling for smoking and other lifestyle factors [1]. Individuals with lower educational attainment may face barriers to accessing healthcare services, including screening and early diagnosis [2], which can lead to more advanced disease at the time of detection. Furthermore, employment status, particularly among those with unstable or manual jobs, has been linked to increased exposure to occupational carcinogens such as asbestos, which further exacerbates lung cancer risk [3]. Understanding how these social determinants interact with clinical risk factors is critical to improving prevention and treatment strategies for at-risk populations.

To reduce lung cancer mortality, early detection is crucial. Lung cancer continues to be the leading cause of cancer-related deaths in both men and women [4]. At present, low-dose computed tomography (LDCT) screening is widely used in several countries, including the United States [5], with many others conducting pilot studies to evaluate the feasibility of lung cancer screening [6]. Additionally, the increased detection of incidental pulmonary nodules (IPNs) has led to more early-stage lung cancer diagnoses [4], contributing to a gradual improvement in overall survival rates [4]. The current U.S. Preventive Services Task Force (USPSTF) eligibility criteria for lung cancer screening recommend screening for individuals aged 50–80 years with a history of at least 20 pack-years of smoking [7]. However, these eligibility criteria do not consider demographic factors like employment status and educational attainment and are largely based on inclusion criteria from the National Lung Cancer Screening Trial (NLST) [8] and the NELSON trial [9]. However, several risk prediction models, such as the Prostate, Lung, Colorectal and Ovarian risk model (PLCOm2012), incorporate factors beyond smoking history and age, including race, chronic obstructive pulmonary disease (COPD), family history, body mass index (BMI), and educational attainment which have been identified as independent risk factors for lung cancer [10].

It is well established that smoking initiation rates are highest among individuals with lower educational attainment, particularly those with only primary or secondary school education, while individuals with college or university degrees are significantly less likely to start smoking. In contrast, the likelihood of successful smoking cessation tends to be higher among those with more advanced education. This pattern has been consistently observed across various countries and healthcare systems, as demonstrated by previous studies [11, 12]. Similarly, education is closely linked with other health-related behaviors. For instance, higher educational attainment is associated with healthier dietary patterns, with individuals possessing higher education levels being more likely to adhere to balanced, nutrient-rich diets, while those with lower education levels tend to have less healthy eating habits. This association has been well documented, including in a Danish cohort study showing a positive relationship between educational level and healthy dietary choices in adulthood [13]. Taken together, these findings highlight how education acts as a key social determinant of health, influencing not only smoking behaviors but also broader lifestyle patterns that may affect disease risk and outcomes.

Previously, a small cohort study found that men and women without a high school diploma had elevated lung cancer incidence rate ratios of 3.01 and 2.02, respectively, compared to those with a college education [14]. A meta-analysis of case–control studies indicated that lower socioeconomic status is associated with an increased odds ratio for developing lung cancer [15]. Similarly, a smaller cohort study found that lower socioeconomic status correlates with a reduced health-related quality of life [16]. In cancer patients, educational level has been shown to be inversely associated with cancer mortality [17]. However, another study found that cancer patients with lower educational attainment who enroll in clinical trials often present with a worse physical condition compared to those with higher education. Interestingly, once enrolled in a clinical trial, educational level did not impact survival outcomes for patients with small cell lung cancer (SCLC) or stage III/IV non-small cell lung cancer (NSCLC). This lack of difference may be attributed to standardized treatment, enhanced patient support, and follow-up protocols inherent to clinical trials [18].

Previous research examining the relationship between lung cancer, educational level, and employment status has largely been based on cohort studies and meta-analyses of such studies. The aim of the current study was to describe the educational attainment and employment status of lung cancer patients, compared to a matched cohort from the general population, using comprehensive nationwide data spanning 24 years from 1994. This approach offers valuable insights into the characteristics of the lung cancer population.

## Patients and methods

### Data sources

The Danish National Health Service provides tax-funded universal healthcare to all citizens [19]. Each resident of Denmark receives a unique ten-digit Civil Registration System (CRS) number at birth or upon immigration, enabling precise individual-level linkage across national registries and lifelong follow-up. For this study, patients aged 18 years or older who were diagnosed with first-time lung cancer between January 1, 1994, and December 31, 2018, were identified using the Danish National Patient Register (DNPR). This administrative registry has offered complete nationwide coverage for all non-psychiatric diagnoses since 1978 [20]. Reporting to the DNPR is mandatory and submitted by treating physicians, continually monitoring hospital and health service use to facilitate billing. Additionally, the DNPR includes cancer records provided by the Danish Cancer Registry, which has maintained nearly complete coverage of cancer diagnoses in Denmark since 1943 [21].

Diagnoses, both primary and secondary, are classified according to the International Classification of Diseases (ICD) [22]. Lung cancer cases were identified using ICD-10 codes C33-C34. The CRS number was used to gather information on patients’ age and sex. To assess temporal changes, the study population was divided into two periods: 1994–2007 and 2008–2018.

A comparison cohort was established at a 1:4 ratio, with individuals randomly selected from the general population matched on age, sex, municipality, and marital status. Individuals with a lung cancer diagnosis were excluded from this cohort. Data on educational attainment were acquired from the Danish Registry of Education and employment status from the Danish Employment Registry.

### Statistical analyses

Differences in the distribution of educational attainment and employment, and odds ratios were analyzed using logistic regression. Odds ratios are showed with 95% confidence intervals (CI). All statistical analyses were performed using SAS 9.4 TS Level 1M5 (SAS Institute Inc., Cary, NC, USA), and graphical presentations were produced in Microsoft Excel, version 16.71.

### Ethical considerations

Data from the DNPR were analyzed without access to personally identifiable information, ensuring the privacy and confidentiality of individuals included in the registry. Data access is approved by the Research Directory of Region of Southern Denmark.

## Results

During the study period from 1994 to 2018, a total of 109,940 patients were diagnosed with lung cancer. The average age of the patients was 69.7 years, with 54% being male. Table 1 provides a detailed overview of the sex and age characteristics for the two periods: 1994 to 2007 and 2008 to 2018. Furthermore, a comparison cohort from the general population was established at a 1:4 ratio, consisting of 439,542 subjects.

**Table 1:** Baseline characteristics of lung cancer patients

Variable	1994–2007	2008–2018
Lung cancer patients (*n*)	56,071	53,869
Males/females (%)	56.0/44.0	50.8/49.2
Median age (IQR) at diagnosis, years	69 (15)	71 (14)
Age groups		
< 50 years (%)	4.9	2.6
50–59 years (%)	15.7	11.4
60–69 years (%)	30.4	30.7
70–79 years (%)	34.5	36.1
80 + years (%)	14.5	19.2

**Fig. 1:**
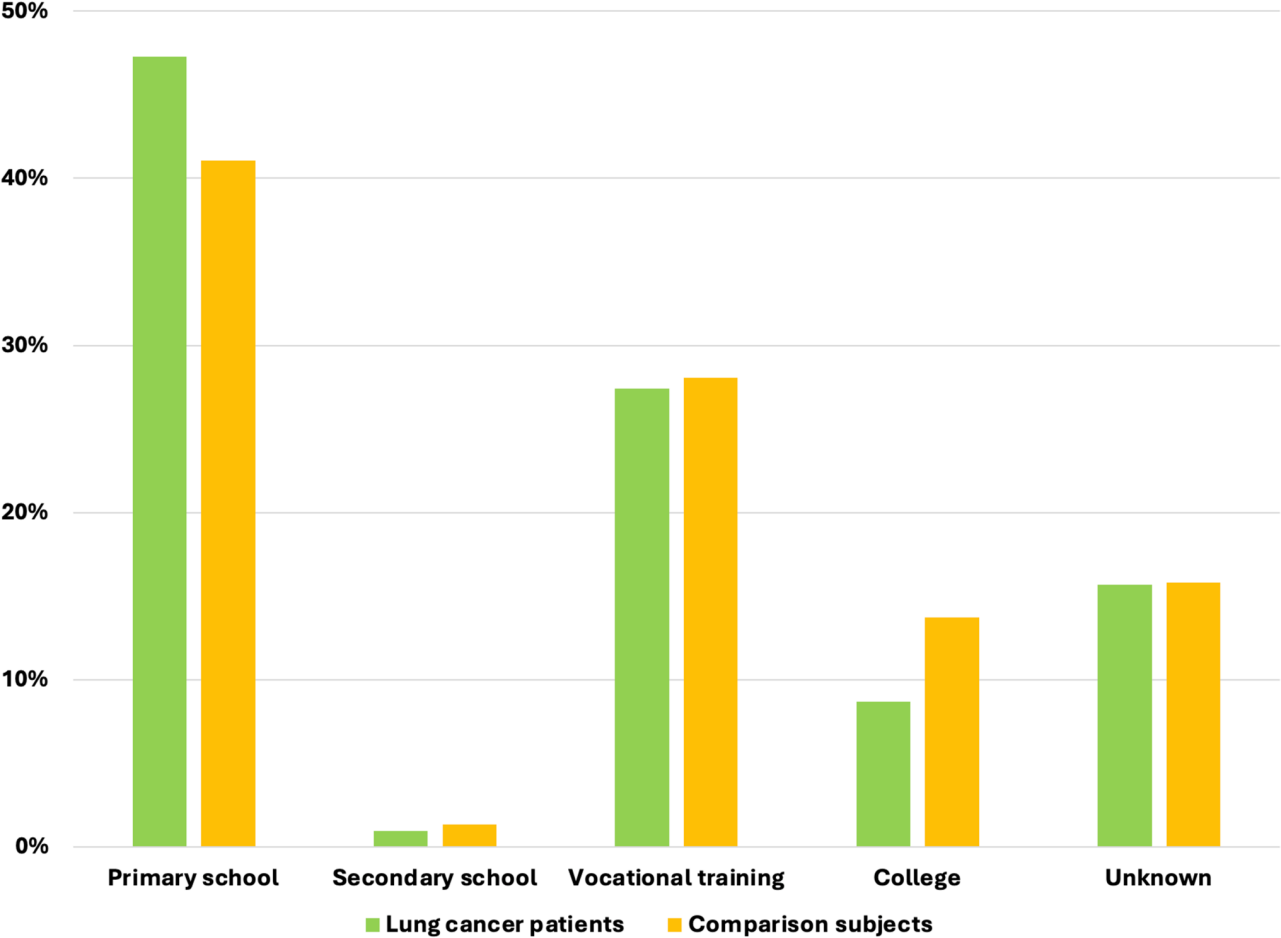
Educational attainment among lung cancer patients and comparison subjects 1994–2007

**Fig. 2:**
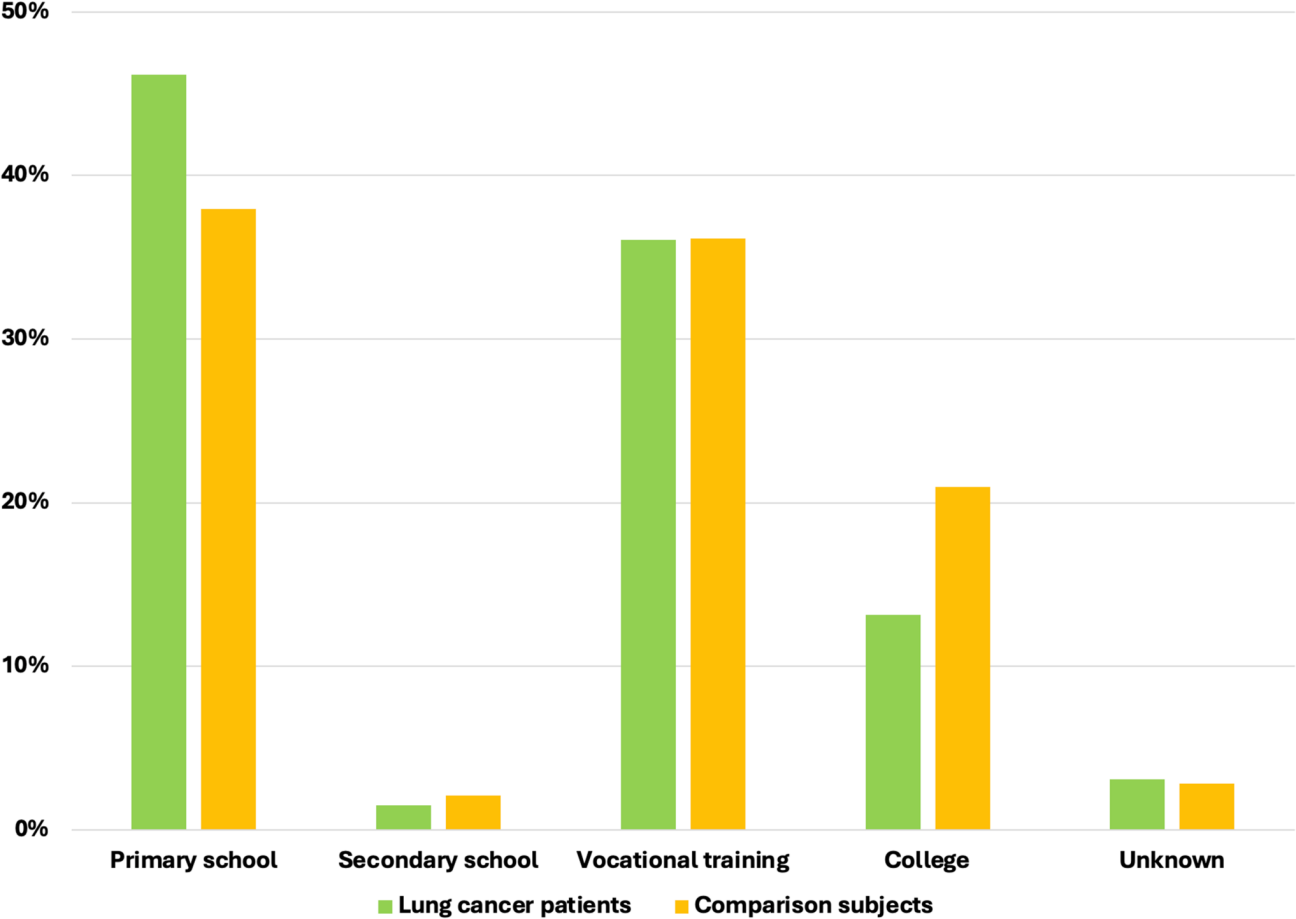
Educational attainment among lung cancer patients and comparison subjects 2008–2018

Among lung cancer patients diagnosed between 1994 and 2007, 47.3% had a maximum educational attainment level of primary school, compared to 41.1% in the comparison cohort (*p* < 0.01) (Fig. 1). This disparity widened in the period from 2008 to 2018, with 46.2% of lung cancer patients having only a primary school education, while this was true for just 38.0% of the comparison group (*p* < 0.01) (Fig. 2).

From 1994 to 2007, 8.7% of lung cancer patients had a college education, compared to 13.2% in the comparison cohort (*p* < 0.01) (Fig. 1). In the following years, the general population reached a higher educational level, and fewer patients had an unknown educational level. However, the gap did not narrow; from 2008 to 2018, 13.7% of lung cancer patients had a college education, as opposed to 21.0% in the comparison cohort (*p* < 0.01) (Fig. 2).

Compared to individuals who had only completed primary school, the odds ratios for developing lung cancer were 0.59 (95% CI 0.54–0.63) for high school graduates, 0.82 (95% CI 0.80–0.84) for those with vocational education, and 0.52 (95% CI 0.50–0.53) for college graduates.

**Fig. 3:**
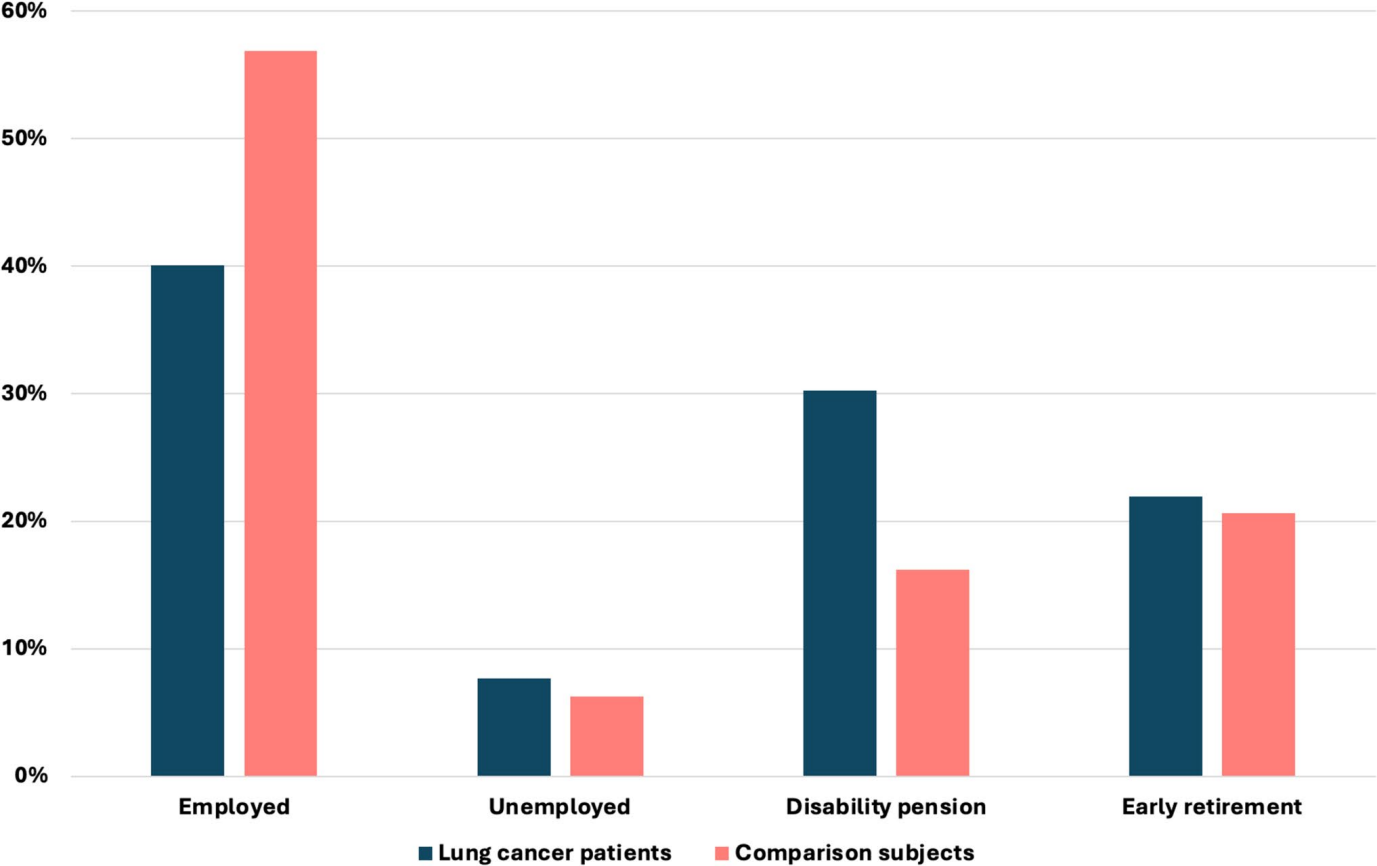
Employment status among lung cancer patients and comparison subjects 1994–2007

A large proportion of lung cancer patients was on age pension due to their age. With the median age of lung cancer diagnosis increasing, the percentage of patients on age pension rose from 59.3 to 69.8% between the two time periods. For those not yet eligible for age pension, employment status between 1994 and 2007 is shown in Fig. 3. The most significant difference was that 40.1% of lung cancer patients were employed, in contrast to 56.9% of the comparison group, representing 42% higher employment rate in the comparison cohort (*p* < 0.01). Additionally, 30.2% of lung cancer patients were on disability pension, compared to only 16.2% in the comparison group (*p* < 0.01). The proportions of individuals who were unemployed, on early retirement, or on ordinary retirement were similar between the two groups.

**Fig. 4:**
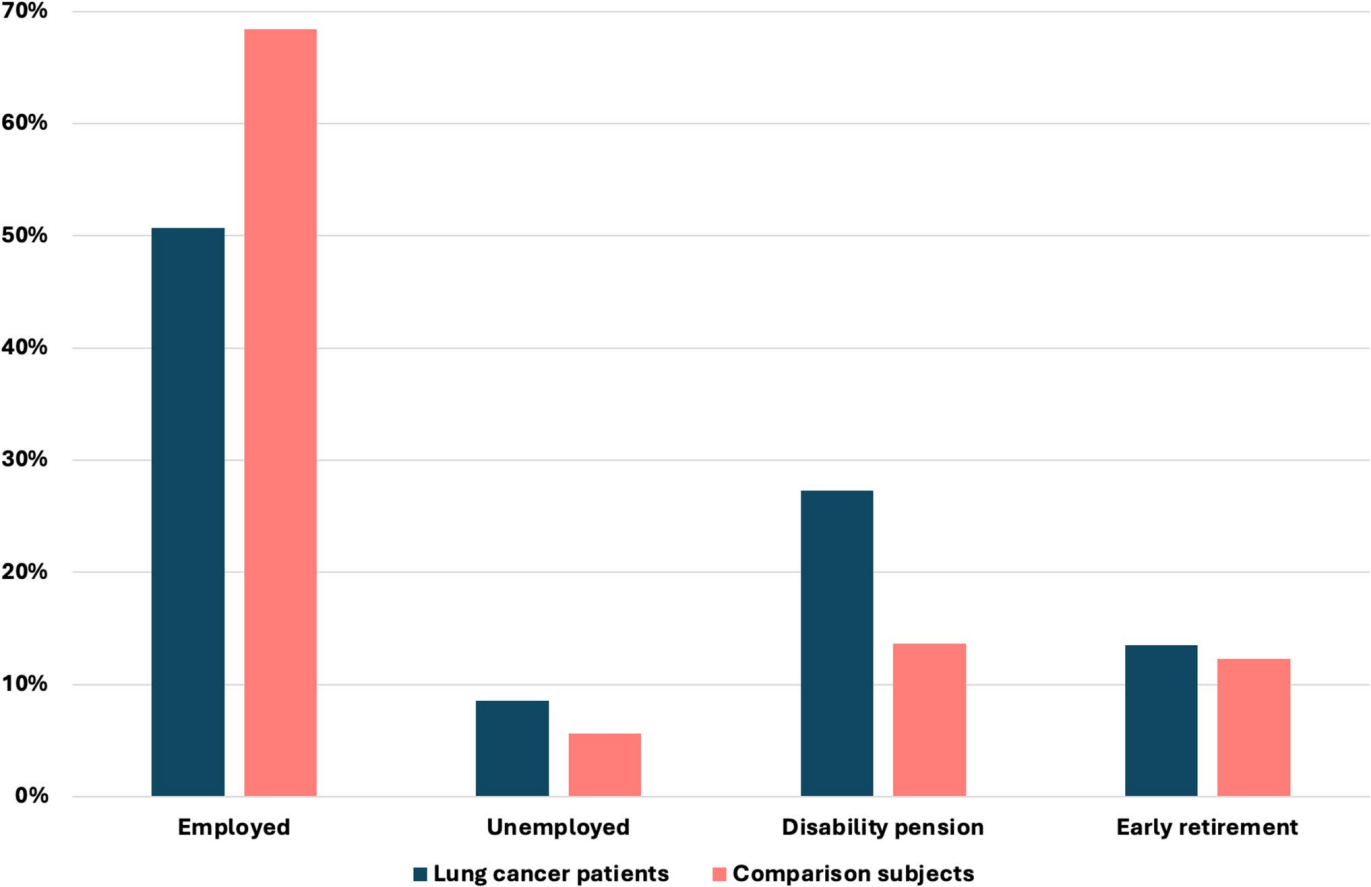
Employment status among lung cancer patients and comparison subjects 2008–2018

Figure 4 presents the employment status of lung cancer patients and comparison subjects from 2008 to 2018. The differences remain largely unchanged; lung cancer patients continue to be more likely to be on disability pension, while comparison subjects are more likely to be employed. Notably, there is a 10% reduction in the number of individuals on disability pension and a 40% reduction in those on early retirement in both groups.

## Discussion

The present study provides important insights into the socioeconomic disparities in the Danish nationwide lung cancer incidence over a 24 year period from 1994 to 2018. We analyzed educational attainment and employment status among lung cancer patients compared to a matched comparison cohort. The findings underscore persistent and significant differences in lung cancer rates according to educational and employment status, highlighting the potential role of socioeconomic factors in disease risk.

Our findings demonstrate a clear and persistent educational disparity between lung cancer patients and the comparison cohort. Across both study periods from 1994 to 2018, patients with lung cancer were more likely to have completed only primary school and less likely to hold a college degree compared to the general population. This gap widened over time, as educational attainment improved in the broader population but remained low among lung cancer patients. These results are consistent with prior studies showing that lower education is associated with a higher risk of lung cancer. One recent study reported that lower educational attainment was linked to increased age-adjusted cancer incidence in men, but not in women, with significant associations for colon, rectal, and lung cancer in men, and for lung and breast cancer in women [23]. Similarly, a Spanish study found that between 1992 and 2003, male lung cancer patients with lower education had consistently higher mortality, while no clear association was observed among women [24]. These differences likely reflect long-standing socioeconomic inequalities in smoking behavior, with smoking rates declining earlier among men than women in several European countries, influencing the observed sex-based disparities [25].

In addition to its association with smoking [11, 12], educational attainment is closely linked with other health-related behaviors and risk factors for lung cancer. Individuals with lower education levels are not only more likely to smoking but also tend to have poorer dietary habits [13], lower levels of physical activity [26], and limited access to health information [27], which can compound their overall disease risk. Furthermore, awareness of early lung cancer symptoms and participation in preventive health services is typically lower in these populations [28]. This clustering of risk factors and health inequalities may contribute to the persistent socioeconomic disparities in lung cancer incidence observed in this study. Addressing smoking alone is unlikely to eliminate these inequalities without considering the broader social determinants of health and health behaviors associated with educational attainment.

The results indicate that health information campaigns and smoking cessation programs should focus on individuals with lower educational attainment, particularly those whose highest level of education is primary school. Targeting smoking behaviors in primary school students, before the onset of pulmonary disease, presents a crucial opportunity. Health promotion efforts should be specifically tailored to socioeconomically disadvantaged communities to ensure effective engagement [29], as smoking is more prevalent in lower socioeconomic groups [30]. Tailoring these programs to such populations has been shown to yield better outcomes than standard smoking cessation programs [31].

While several studies have explored the ability of lung cancer survivors to return to work [32, 33], less is known about their employment status at the time of diagnosis. The current analysis highlights the socioeconomic vulnerability of lung cancer patients. In both study periods, lung cancer patients were less likely to be employed and more likely to be on disability pension compared to the control group. Between 1994 and 2007, only 40.1% of lung cancer patients were employed, compared to 56.9% of the comparison group, with lung cancer patients being twice as likely to receive disability benefits. This trend continued from 2008 to 2018, although there was a decline in disability pension and early retirement rates across both groups. These findings align with the previous finding that unemployment is associated with a higher smoking prevalence [34]. Another finding is that smoking prevalence tends to be higher in stressful occupations [35], which are more commonly linked to disability pensions [36]. This may help explain the observed correlation with lung cancer incidence.

Despite an increase in overall wealth in society over the past few decades, this study demonstrates that such economic growth has not improved the social inequalities in lung cancer incidence. Therefore, increased national wealth alone is insufficient to mitigate this disparity. Notably, in recent years, the incidence of lung cancer among Danish women has reached the same level as that of men, indicating that women are now equally at risk. This trend likely reflects historical patterns of smoking behavior, where female smoking prevalence only approached that of men within the past 15 years, having previously been considerably lower [25]. It is possible that women may also have a lower biological threshold for tobacco-related lung damage compared to men, further contributing to this convergence in incidence rates.

Primary prevention strategies, such as promoting smoking cessation, should specifically target at-risk populations. Additionally, early detection methods, including LDCT screening, must incorporate strategies to engage participants from lower socioeconomic groups effectively. These findings emphasize the importance of targeted public health interventions focusing on reducing smoking rates, increasing health literacy, and providing supportive services to vulnerable populations. These measures are critical for mitigating the disparities and improving lung cancer outcomes.

### Limitations

Overall, the Danish registries are remarkable due to their national coverage, which sets them apart on a global scale. They capture all lung cancer diagnoses, and their integration with other comprehensive registries enhances their uniqueness. However, during the initial period from 1994 to 2007, coverage of educational attainment was only 85%. This limitation affects both groups and is believed to be evenly distributed, minimizing the risk of bias in comparisons between the two cohorts.

While the findings offer valuable insights, they may not be applicable to countries with different public support systems and lung cancer population characteristics, potentially limiting their broader applicability. Despite the comprehensiveness of the registries, there may still be instances of missing data or inaccuracies in the recorded information, which could impact the reliability of the analysis.

## Conclusion

In conclusion, this study demonstrates significant and persistent socioeconomic disparities in lung cancer incidence, with individuals of lower educational attainment and those on disability pension being at higher risk. This underscores the need for primary prevention and early detection strategies to specifically focus on these at-risk populations.

## Data Availability

Anonymized data will be provided by the corresponding author at reasonable request after approval of Statistics Denmark Database.
